# Axial rotation affects the cognitive characteristics of spatial ability

**DOI:** 10.3389/fpsyg.2024.1396441

**Published:** 2024-10-29

**Authors:** Tian Feng, Fuchun Zhang, Jinzhao Liu, Manqi Liang, Yawei Li

**Affiliations:** ^1^Department of Sports, Henan Sport University, Zhengzhou, China; ^2^School of Physical Education, Zhengzhou University, Zhengzhou, China; ^3^Department of Physical Education, Henan Sport University, Zhengzhou, China

**Keywords:** mental rotation, sport type, axial rotation, sex, age

## Abstract

**Purpose:**

To test spatial ability in athletes with different axial rotation experience and analyze their behavioral data to explain the cognitive mechanisms of spatial ability in athletes.

**Methods:**

Experiment 1: A total of 147 athletes were selected for the paper-and-pencil mental rotation test (MRT). The athletes were separated according to three sport types: open high-spatial (OH) sport, closed high-spatial (CH) sport, closed low-spatial (CL) sport. Spatial ability testing with a two-factor mixed experimental design of 3 (sport type) × 2 (stimulus type). Experiment 2: In this study, 47 players were selected for computerized mental rotation test, with a three-factor mixed experimental design of 3 (sport type) × 2 (angle: 45°, 90°) × 3 (rotational axis: left–right axis, up–down axis, and front–back axis). Repeated-measures ANOVA was performed to evaluate the data.

**Results:**

(1) The CH group and OH group outperformed the CL group in the non-embodied task (all *p*s < 0.003) and the CH group was better than the other groups in the embodied and tasks (all *p*s < 0.008). (2) Under 45° rotational conditions, the reaction time (RT) for the left–right (LR) and up–down (UD) axes were shorter than that for the front-back (FB) axis (all *p*s < 0.026). However, under 90° conditions, the RT for FB < LR < UD, with superior accuracy and rotational speed for the FB axis than for the LR and UD axes (all *p*s < 0.034). (3) Male players from the CH and CL groups had shorter RTs than did those from the OH group at both angles (all *p*s < 0.047). For female players, the CH group presented a shorter RT than the OH and CL groups did at 90° (all *p*s < 0.006). (4) No sex difference was found for paper and pencil MRTs, but a male advantage existed only in the CL group for computerized MRTs (*p* = 0.005).

**Conclusion:**

The motor skills associated with axial rotation could promote mental rotation performance and compensate for sex differences in mental rotation ability.

## 1 Introduction

### 1.1 Mental rotation

Mental rotation (MR) is a type of intelligence in the spatial cognitive system and refers to the ability to generate, retain, extract, and transform visual images (Wai et al., 2009). Only with good mental rotation ability can we learn and complete precise operations when dealing with complex environments, which is necessary not only in daily life but also in the process of learning and performing movement skills. Therefore, mental rotation is important for the psychological development of elite individuals.

#### 1.1.1 Transformations

Mental rotation consists of two different classes of representations according to the reference frame of rotation: object-based representation and egocentric transformation (Zacks et al., 2002). In the former, subjects perform rotation operations from the third-person perspective, such as watching the leftward movement. In the latter, subjects rotate themselves from a first-person perspective, such as imagining themselves making a leftward turn. According to previous studies, same–different judgments about pairs of pictures are often required for object-based representation and left–right judgments about single pictures for egocentric transformation (Zacks et al., 2000). Voyer et al. (2017) investigated the effect of stimulus type (embodied: hand and non-embodied: letter) on egocentric (left–right judgment) or object-based (same-different judgment) processing in mental rotation and demonstrated that the mental rotation slope for response time was steeper for object-based than for egocentric transformations, verifying different patterns of these two kinds of mental rotation.

#### 1.1.2 Measurement methods

The classical mental rotation test was designed by Shepard and Metzler (1971). Scholars have conducted a variety of experiments based on this paradigm from different perspectives. However, Jansen reported that different measurements can also affect the results (Jansen et al., 2012). Compared with paper and pencil tests, computerized MR tests have been conducted in recent years, which regularly report better performance for men (Debarnot et al., 2013; Peters et al., 2007). Moreover, the number of possible alternatives and whether they are presented as pairwise mirrors are often employed, and a novel study has shown that the overall performance is lower for more alternatives and for mixed alternatives but not for their interaction, suggesting that the differences between tests affect performance (Jost and Jansen, 2023).

#### 1.1.3 Angle effect

As most researchers have shown (Jola and Mast, 2005; Shepard and Metzler, 1971), RTs increase gradually as a function of angular disparity, which means that the more the angle is rotated, the more time the participants are required to react. This may account for the cognitive load at different angles. The cognitive load is one of the factors that influences mental rotation ability (Morawietz and Muehlbauer, 2021). Bennett reported that as the cognitive load increases, participants’ accuracy in working memory tasks decreases (Bennett et al., 2013). Studies have confirmed that as interference (i.e., large angular disparity) intensifies, participants’ processing of information decreases (Gignac and Vernon, 2003; Bauer et al., 2022).

#### 1.1.4 Individual differences

Studies have suggested that there is at least one sensitive period in the development of adolescents’ MR ability (Quaiser-Pohl et al., 2014). MR ability develops rapidly after the age of 10 years and tends to mature around the age of 14–17 years (Doerr et al., 2021; Rahe et al., 2018; He et al., 2019) but gradually decreases with age after the age of 20 years (Borella et al., 2014). In terms of adolescents, middle school students can accurately complete mental rotation tasks (Neuburger et al., 2011). Therefore, adolescent athletes were chosen as the subject of this study.

Additionally, sex differences have been widely studied in the field of MR. Studies have shown that the spatial cognition ability of males is greater than that of females (Hegarty and Waller, 2005). The researchers compared the mental rotation ability of 42 males and 42 females and reported that sex was an effective factor in predicting mental rotation test scores; that is, males had stronger mental rotation ability than females did. One study compared the spatial ability of adolescents with expertise in STEM, arts, and sports with that of their unselected peers to assess sex differences across expert groups. They reported that sex differences persisted in all expert groups (Tsigeman et al., 2023). The reason may be that men are more likely to use the global-shaped strategy and more holistic strategies than women are (Hegarty, 2018). However, when the stimulus type was changed from letter to hand, a reduced male advantage for egocentric rotations compared with object-based rotations was found (Voyer et al., 2017).

### 1.2 Motor skill learning and mental rotation

#### 1.2.1 Embodied MR for motor experts

As a type of representational operation, mental rotation involves similar cognitive processing to that of real rotation (Wohlschläger and Wohlschläger, 1998). A previous study revealed that the motor areas of the brain are also activated when individuals mentally rotate (Jordan et al., 2002). Therefore, mental rotation ability is closely related to motor expertise (Jost et al., 2023). Some studies have compared the mental rotation ability of individuals with different degrees of sports experience and reported that the mental rotation ability of expert athletes is better than that of ordinary people (Moreau et al., 2015; Schmidt et al., 2015; Moreau et al., 2011). Athletes manipulate and move their bodies in space to practice and improve their skills. Therefore, the advantage for athletes in mental rotation ability after years of training is actually a process of embodiment. In light of embodied cognition, cognitive processing is based on the physical state (Wilson, 2002; Gallese and Sinigaglia, 2011), and all types of cognitive activities, such as thinking, classification and judgment, are not just information processing in the brain but are closely related to one’s physical properties, perception, and movement ability.

#### 1.2.2 Sport type

Previous studies have shown that the embodied MR ability of athletes is moderated by sport type. Different sports have different effects on mental rotation ability. Studies have shown that gymnastics and orienteering participants have better mental rotation abilities than soccer and running participants do (Jansen and Lehmann, 2013; Schmidt et al., 2015). Accordingly, it has been further suggested that the stronger the correlation between mental rotation task stimuli and sports experience is, the better the athletes’ performance and that the mental rotation ability of athletes in different sports shows “selective influence” (Feng et al., 2017). According to the perspective of action imitation theory, some mental representations and internal mechanisms are shared between motor representations and action execution (Barsalou, 2008). After training, athletes’ nervous system can coordinate with sensory input, thus accelerating the perception of their surroundings and increasing the efficiency of the use of psychological resources (Yin, 2024). However, some studies concerning the spatial essentials of events fail to elucidate the differences between high-spatial sport and low-spatial sport. Sylvie et al. (2002) used two-dimensional cube MRTs to compare sports with high rotation requirements (gymnastics) and low rotation requirements (handball, rugby, basketball, football, badminton, wrestling, judo, track and field), Habacha et al. (2014b) investigated the MR performance of spatial (football, handball, basketball, racket, hockey, gymnastics) and non-spatial (track, wrestling, and swimming) players in three-dimensional MRTs, and Sylvie et al. (2004) examined the MR ability of open sports (rugby, basketball, soccer, badminton, wrestling, judo and tennis) and closed sports (medium distance running, cycling, swimming, gymnastics, archery, javelin, and fitness). However, these methods did not distinguish by different spatial sport types, indicating that the classification method may need to be modified.

#### 1.2.3 Task stimuli

As mentioned previously, stimulus type (i.e., rotation side, direction and angle) can strongly influence the performance of motor experts (Habacha et al., 2014c; Habacha et al., 2014a). Researchers reported that table tennis players made judgments faster on the dominant hand than on the nondominant hand in a mental rotation task (Habacha et al., 2014c). The reactions of gymnasts were faster than those of nonathletes only in their dominant rotation direction (Habacha et al., 2014a). Divers were faster than nonathletes were in judging images at an inverted angle of 180° and showed differences in brain activity (Feng and Li, 2021). In addition, another vital index of rotational movements is the axis. Using the spatial coordinate system as a reference, the axes of human rotation can be divided into the left–right axis, the up–down axis, and the front–back axis. Fargier et al. (2022) compared the MR performance of futsal and rhythmic gymnasts and sedentary people and reported that the reaction time of the football group was shorter in the frontal, horizontal, and sagittal planes than that of the sedentary group, and their reaction time in the horizontal plane was also shorter than that of the rhythmic gymnastics. However, in terms of the MR ability of motor experts with different practices in the rotation axis, whether athletes who are good at rotating their body with multiple axes, such as aerial freestyle skiing athletes, outperform other athletes who often rotate with a single body axis remains unclear.

### 1.3 The current study

In summary, mental rotation is important for the psychological development of elite individuals, but object-based and egocentric transformations of MR had different patterns of cognitive processing. Individual differences can affect mental rotation ability. Specifically, adolescence is a critical period for the development of mental rotation ability (Doerr et al., 2021; Rahe et al., 2018; He et al., 2019), and males are often considered to have better mental rotation abilities (Hegarty and Waller, 2005). Building on this, from the perspective of embodied cognition, the present study wants to examine the relationship between motor skill learning and spatial ability to address a series of important questions. First, because some studies concerning the spatial essentials of events fail to found the differences between high-spatial sport and low-spatial sport, what role the spatial characteristics of the sport play in the effect of sports experience on mental rotation ability. Can this role of spatial characteristics in sports interact with the stimulus characteristics in mental rotation tasks (such as angle and rotation axis)? In addition to this, can motor skill learning further enhance the mental rotation ability of adolescents, or can it compensate for gender differences in mental rotation ability? Therefore, to investigate the effect of motor expertise type on MR, the spatial factor of sports training, which is coupled with the characteristics of the task stimuli, especially the material angle and axis, should be examined with open-closed types of sports. The present study classifies the spatial sport type via the matrix of high-spatial/low-spatial and open/closed types. According to previous research, closed sports include a stable environment and predictable events (i.e., swimming), but open sports require fast and frequent reactions to unpredictable environmental changes (i.e., basketball) (Sylvie et al., 2004), and high-spatial sports are events that involve mental and physical rotations in their practice (i.e., gymnastics). Low-spatial sports refer to activities that require very little motor rotation (i.e., track and field) (Sylvie et al., 2002). The present study divided three kinds of sport: (1) open high-spatial sport (basketball, soccer, and tennis), (2) closed high-spatial sport (Tai Chi, wrestling, gymnastics, aerobics, aerial freestyle skiing) and (3) closed low-spatial sport (running). Because open sports often involve complex spatial transformation, open low-spatial sports are not defined. Two experiments were conducted to test the influence of axial rotation experience on spatial ability, as well as their interaction with MR transformations, measurement methods and individual differences (age and sex). Experiment 1 used paper and pencil tests to verify the spatial ability differences among the three types of adult athletes, and we hypothesized that the open high-spatial group and the closed high-spatial group would outperform the closed low-spatial group in MRTs with both embodied and non-embodied materials. On this basis, experiment 2 conducts computerized MR tests with egocentric transformations to investigate the spatial ability differences among teenagers in the three types of sports. Similarly, we hypothesized that the open high-spatial and closed high-spatial groups would be superior to the closed low-spatial group and that their advantages would be shown in certain axes or angles congruent with their training experience.

## 2 Experiment 1

### 2.1 Participants

A total of 147 participants were enrolled and divided into three groups: (1) open high-spatial sport (OH), which included 50 players from basketball, soccer, and tennis and 26 males and 24 females aged 19 years (68 ± 0.74 years). Their training period was 3.00 ± 2.00 years; (2) closed high-spatial sport (CH), which included 50 players from Tai Chi, wrestling, gymnastics, aerobics, 26 males and 24 females, aged 18.78 ± 1.49 years, and their training period was 2.19 ± 1.49 years; (3) closed low-spatial sport (CL), which included 47 players from running, 28 males and 19 females, aged 19.72 ± 0. 93 years, and their training period was 4.19 ± 2.41 years. A power analysis of the RM ANOVA was conducted with G*Power software using the following settings: expected effect size of 0.25, α-level of 0.05, sample size of 144, and power of 0.99 (1−β). We recruited participants at the Physical Education College of Zhengzhou University and gave their informed consent to participate in this study. None of the participants had completed a mental rotation test before or had been trained in mental rotation.

### 2.2 Stimuli and task

**Figure 1:**
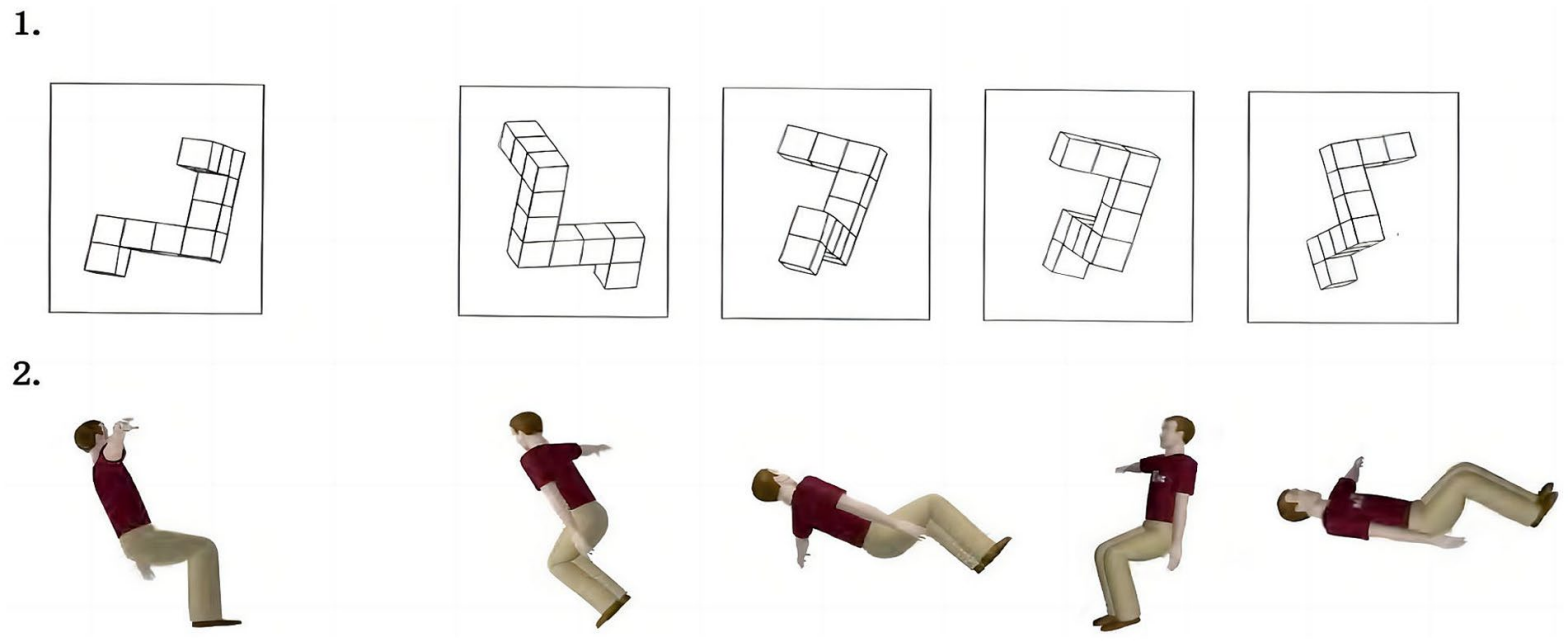
Example of the mental rotation test in Experiment 1.

This study used a modified mental rotation test (MRT) developed by G. Alexander of Texas A&M University (Alexander and Evardone, 2008) to investigate mental rotation ability, as shown in Figure 1. The test contains 24 questions, including 12 original non-embodied (cube) items from the Vandenberg and Kuse (1978) mental rotations test (Peters, 2005), which has been used extensively in the experimental literature on spatial ability. The other 12 embodied (body) figures, constructed via Autodesk Maya 6.5 software, depicted three-dimensional males and females dressed in t-shirts and pants. In three-dimensional space and for the serial positioning of correct items and distracters. Each question has one reference graph and four optional graphs. The participants are required to determine which two of the four options are derived from the rotation of the reference graph. The authors have permission to use this instrument from copyright holders.

### 2.3 Procedure

The study was conducted in a quiet laboratory room at Henan Sport University on 16 June 2020. Each subject was individually tested. A questionnaire referring to their individual information (including sex, age, exercise level, number of training years, best performance, etc.) was used. Next, they took part in a pencil and paper MRT. The MRT consisted of 24 questions, averaged across four sheets of paper. The instructions for each measure were read aloud, and the participants were provided the same amount before the test began. The participants constructed two practices to ensure that they understood the test and that the practical questions did not appear in the formal test. After the practice period, the participants were asked to choose the two alternatives. As in earlier research (2008), they were asked to complete the first 12 questions in 3 min, followed by a 2-min midway break, and finally, they were asked to complete the last 12 questions. The entire experiment was supervised by two experimenters. Only when the two correct choices of the target figure are correctly selected will the subject receive a point. The participants could score a maximum of 24 points: 12 points for the human body and 12 points for the cube.

### 2.4 Data analysis

The MR scores for every group obtained a normal distribution (*z* < 1.900, *p* > 0.166, in all instances). Repeated-measures analysis of variance (R-M ANOVA) for MR scores was performed with the between-subject factors of groups (OH, CH, CL) and sex (male, female) and the within-subject factor of stimulus type (cube, body). When the interaction was significant, simple effects analysis was conducted, and the Bonferroni correction method was used for correction. *p* < 0.05 indicates statistical significance, and the partial Eta square (*η*^2^) represents the effect size of the analysis of variance.

### 2.5 Results

**Figure 2:**
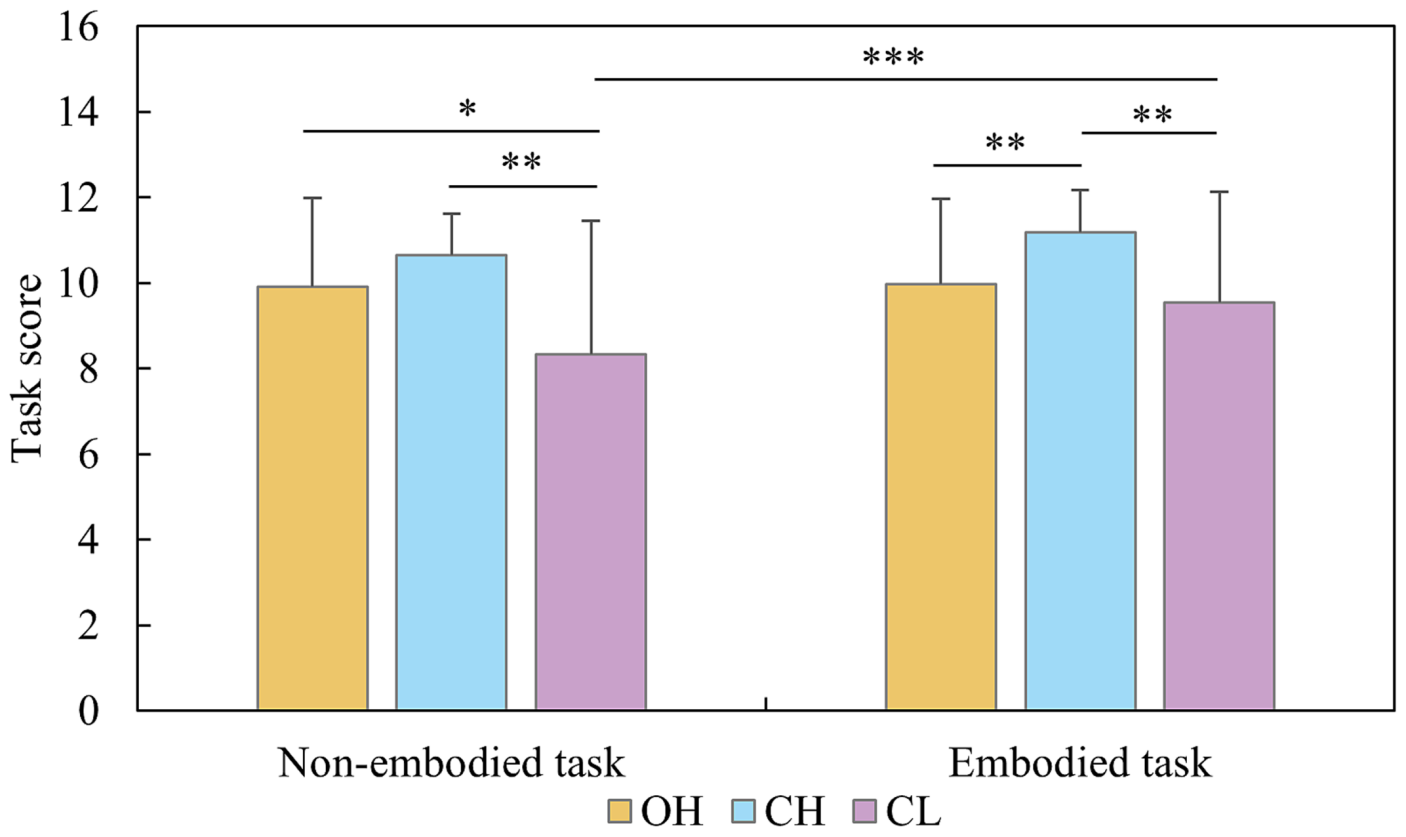
Scores on mental rotation tests for different groups and stimulus types (*p < 0.05, **p < 0.01, ***p < 0.001).

Analysis of the MR score revealed a significant main effect of group, *F* (1, 141) = 12.532, *η*^2^ = 0.093, *p* < 0.001, and stimulus type, *F* (1, 141) = 14.494, *p* < 0.001, *η*^2^ = 0.151, but not for sex, *F* (2, 141) = 0.005, *p* = 0.945, *η*^2^ < 0.001. The interaction effect between group and stimulus type was significant, *F* (2, 141) = 4.458, *p =* 0.013, *η*^2^ = 0.059. Simple effects analysis revealed that in the non-embodied cube task, the OH group (9.92 ± 2.06, 95% CI [9.29, 10.54]) and CH group (10.66 ± 0.96, 95% *CI* [10.03, 11.27]) scored significantly higher than the CL group (8.34 ± 3.12, 95% *CI* [7.74, 9.04], *p* < 0.003, for all instances). In the embodied body task, the CH group (11.18 ± 1.00, 95% *CI* [10.63, 11.73]) performed better than did the OH (9.98 ± 1.98, 95% *CI* [9.42, 10.52]) and CL groups (9.55 ± 2.59, 95% *CI* [9.04, 10.19], *p* < 0.008, for all instances). When the scores of stimulus type for each group were compared, the results revealed that only the CL group had different scores between the cube (8.39 ± 3.12, 95% *CI* [7.73, 9.04]) and body tasks (9.62 ± 2.59, 95% *CI* [9.04, 10.19], *p* < 0.001; Figure 2).

### 2.6 Discussion

In Experiment 1, a classical pen-and-paper assessment was employed to examine disparities in spatial ability among three distinct categories of adult athletes. A total of 147 athletes from open high-spatial sport (basketball, soccer, and tennis), closed high-spatial sport (Tai Chi, wrestling, gymnastics, aerobics) and closed low-spatial sport (running) sports were selected and participated in the experiment. Overall, the results showed that the CH group and OH group outperformed the CL group in the non-embodied task and the CH group was better than the other groups in the embodied and tasks. No sex difference was found for paper and pencil MRTs.

First, differences between the two types of stimuli were confirmed. Embodied cognition suggests that cognitive processing is based on the physical state (Wilson, 2002; Gallese and Sinigaglia, 2011). With respect to mental rotation, body stimuli could facilitate individuals’ embodied processing, revealing the common cognitive component between mental rotation and motor rotation; thus, participants in the CL group answered more questions about body stimuli than the cube stimuli. Moreover, the embodied figure task often seems to lead to egocentric transformation of mental rotation (Feng et al., 2017), in which sport expertise facilitates performance because the human body stimulus elicits embodied spatial transformations (Kaltner et al., 2014), even though some studies have suggested that this transformation exists mainly in left–right judgments about single pictures (Zacks et al., 2000).

Concerning the effect of motor expertise on spatial ability, we hypothesize that participants categorized into either the OH group or the CH group are expected to demonstrate superior performance than the CL group on MRTs, irrespective of whether the materials involved are embodied or not embodied. The result showed that the CH group and OH group outperformed the CL group in the non-embodied task and the CH group was better than the other groups in the embodied and tasks. With respect to the result that only the CL group showed different performance between the cube and body tasks, this finding may indicate that sport experience from open skills could ensure the embodiment of body experience in mental rotation. In particular, this effect can be transferred to non-embodied tasks. This may be because open ball sports require athletes to be aware of their teams’ and opponents’ movements at all times during training and competition, including rotational movements (e.g., basketball and football), which require them to change their strategies and plans on the basis of the change in entrance, thus enhancing their object-based transformation ability and making them more similar to athletes in closed high-spatial sports. This result is consistent with the findings of several studies (Sylvie et al., 2004; Pasand et al., 2015; Habacha et al., 2014b). A study by Jansen et al. (2018) investigated whether increased physical education in schools could enhance mental rotation skills. Adolescents were split into two groups: one receiving extensive physical education and the other receiving a standard amount. Both groups completed questionnaires and took a mental rotation test. The group with more physical education significantly outperformed the control group on the test, indicating that additional physical education training led to improved mental rotation ability.

These findings suggest that it is reasonable to compare the mental rotation ability of high-spatial/low-spatial and open/closed sports. Similar comparisons have been made in previous studies, but differences were not found. In a previous study, athletes were divided into sports with high rotation requirements (gymnastics) and sports with low requirements (i.e., handball, football, basketball), wrestling, judo, and athletics. Similarly, Habacha et al. classified sports into spatial (football, handball, basketball, racket sports, hockey, gymnastics) and non-spatial (athletics, wrestling, swimming) sports on the basis of the complexity of spatial factors; however, no differences were found between the groups (Sylvie et al., 2002; Habacha et al., 2014b). There may be two reasons for those results. First, these open high-spatial sports, such as ball games, were treated as non-rotational sports. Second, some closed high-spatial sports, such as wrestling and judo, were also treated as single-axial or non-spatial sports. The results of the study revealed that both closed and open high-spatial sports were able to similarly enhance the mental rotation ability of the athletes.

In addition to sports, sex, an individual factor, has been widely studied in the field of spatial cognition. Studies have shown that the spatial cognition ability of males is greater than that of females (Hegarty and Waller, 2005). However, the effects of motor skill learning on sex differences have not been extensively studied (Voyer and Jansen, 2017). For male and female athletes who have been engaged in sports training for many years and who have similar experience, can sex differences be made up? The conclusions of the current studies are inconsistent. From the perspective of cognitive plasticity, researchers believe that a sex difference between male and female athletes already exists and that the gap between the two is different in the process of accumulating sports experience. Therefore, even if they both have sports experience, a sex gap still exists (Pietsch and Jansen, 2012; Schmidt et al., 2015; Fargier et al., 2022). In contrast, some studies believe that female athletes can achieve greater improvements in activities; that is, sex differences can be compensated for by participation in physical activities (Habacha et al., 2014b). Therefore, the present study investigated the influence of sex on players’ mental rotation, and no sex difference was found within either group or task type, which supported the latter opinion.

## 3 Experiment 2

### 3.1 Participants

The study population, comprising 46 people, was divided into three groups on the basis of the type of sport: (1) an open high-spatial sport (OH) group consisting of 16 basketball and soccer players (8 males and 8 females) who were 13.31 ± 0.79 years old and had 2.23 ± 1.43 training years. (2) a closed high-spatial sport (CH) group consisting of 15 freestyle skiing players (8 males and 7 females) who were 14.60 ± 1.02 years old and had 3 ± 0.34 training years; and (3) a closed low-spatial sport (CL) group consisting of 15 runners (9 males and 6 females) who were 14.60 ± 1.78 years old and had a training time of 1.27 ± 0.44 years.

### 3.2 Task stimuli

**Figure 3:**
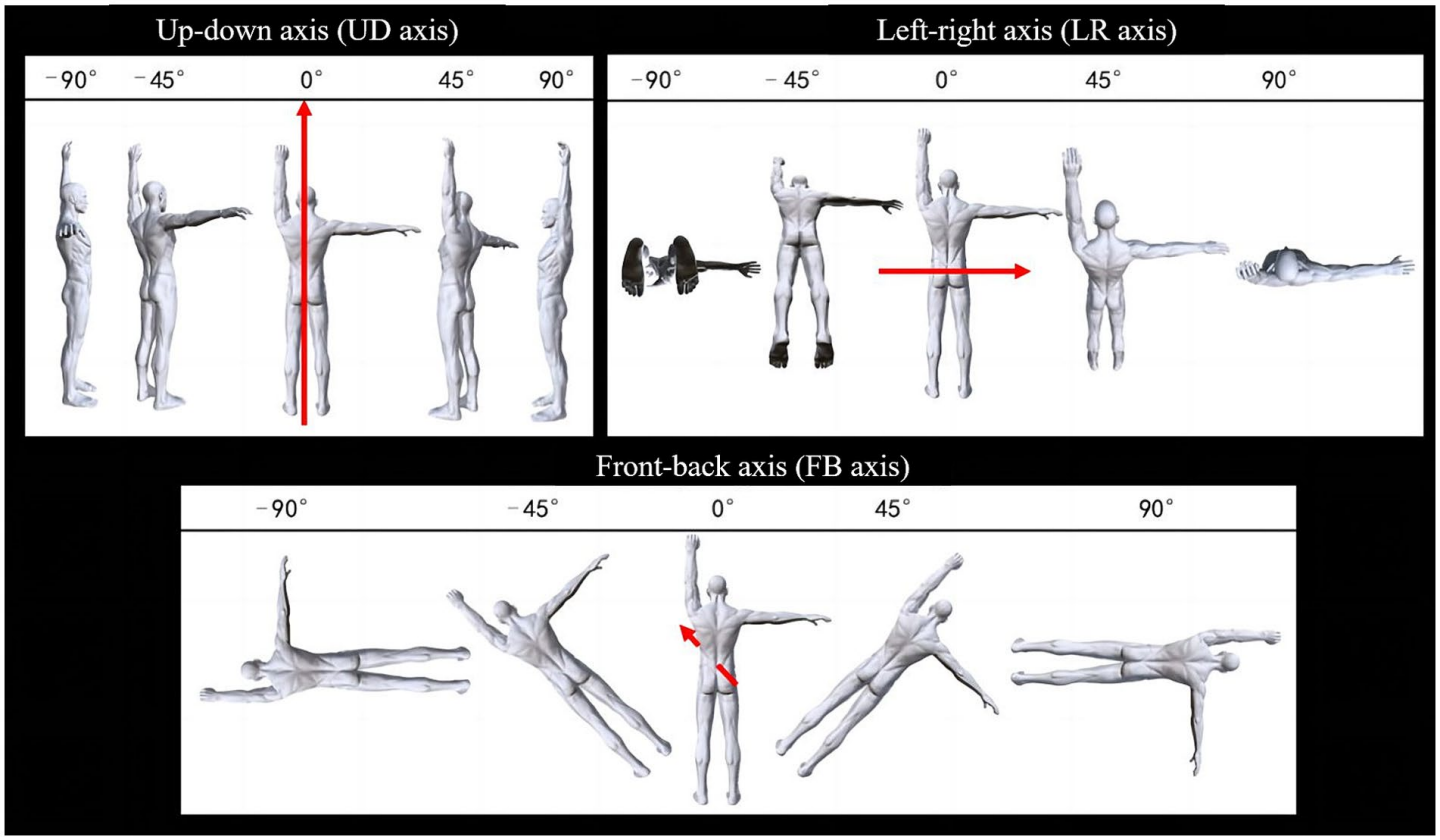
Example of the stimuli used in Experiment 2.

The experimental stimuli were body movement pictures designed with ArtPose software, which consisted of a picture of a male with one arm raised upward and the other arm raised sideways. Only one figure is presented at a time, and the subject needs to choose which arm was, according to the axial characteristics of the movement, three types of rotation are distinguished: (1) rotation around the left–right (LR) axis of the body. (2) Rotation around the up–down (UD) axis of the body. (3) Rotation around the front-back (FB) axis of the body. The rotation angle of each axial direction includes a small angle (45°) and a large angle (90°), as shown in Figure 3. The image size is 700 × 900 pixels. The experimental task was designed with E-Prim 3.0 (Psychology Software Tools, www.pstnet.com) and presented using a 23.8-inch computer.

### 3.3 Procedure

**Figure 4:**
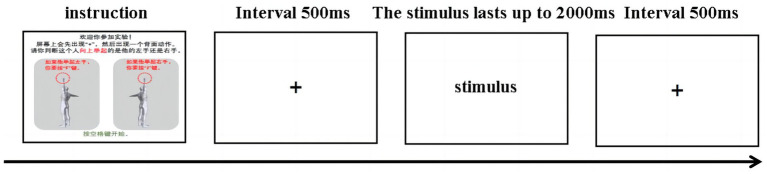
Mental rotation task in Experiment 2.

The experiment was conducted in the laboratory of Henan Sport University, and each subject was tested separately in a closed, quiet, noninterference environment. After the purpose and operation of the experiment were explained, the participants were seated in front of the computer (the eyes were 60 cm away from the screen). The experimental data were prepared via E-Prime 3.0 software. There were six stimulus conditions (left–right axis at a small angle, left–right axis at a large angle, up–down axis at a small angle, up–down axis at a large angle, front-back axis at a small angle, and front-back axis at a large angle). First, all participants attended a practice part with 10 experimental trials. In each trial, 500 ~ 800 ms of fixation occurred in the middle of the screen, after which a stimulus picture was presented. The subjects were required to determine whether the man in the picture was raising up his left arm (press key “F”) or the right arm (press key “J”) accurately and quickly. The picture disappeared after the button or time exceeded 4,000 ms, and a feedback screen (showing “right” or “wrong”) was presented for 500 ms before the next trial. Responses submitted more than 4,000 ms after presentation of the stimulus were considered as errors. The stimulus in practice part included pictures with all combines of axis and angle. The practice accuracy of every subject had to be above 80% to ensure that they had completely understood the task. Two subjects practiced twice to reach the criterion. Second, the formal experiment used similar test trials in practice but did not give feedback. The formal experiment contained three same blocks, each containing 90 randomly ordered trials (for 6 stimulus conditions × 15 repetition). Each subject rested for 10 s before moving on to the next block until all three blocks were completed. The response time and accuracy were obtained. The overall duration of the experiment was approximately 20 min (Figure 4).

### 3.4 Data analysis

The reaction time (RT), accuracy and stage performance were calculated for every group. According to the phases of information processing, mental rotation is subdivided into the perceptual stage, rotation stage, and decision stage (Heil and Rolke, 2002; Shepard and Cooper, 1982; Corballis, 1988). Specifically, the performance in the perceptual and decision stages is the RT when the stimulus is not rotated, and the mental rotation speed represents the performance of the rotation stage. In the present study, the rotation speed was used as the performance of the rotation stage and was the average of the ratio of the angle at each angle to the RT. The calculation formula is $\mathrm{rotational speed}=\left(\frac{45}{RT_{45{}^{\circ}}}+\frac{90}{RT_{90{}^{\circ}}}\right)÷2\times 1000$ , where the unit is represented in degrees per second (°/s). The perceptual time was subsequently calculated via the following formula: RT = perceptual time + angle*rotational speed. Normal distributions were obtained for all variables (*z* < 1.166, *p* > 0.132, in all instances). With respect to RT and accuracy, four-factor R-M ANOVA was conducted with the between-subject factors of group (OH, CH, CL) and sex (male, female) and the within-subject factors of the rotational axis (left–right (LR) axis, up–down (UD) axis, and front–back (FB) axis) and angle (45°, 90°). For stage performance, three-factor R–M ANOVA was conducted with the between-subject factors of group (OH, CH, CL) and sex (male, female) and the within-subject factors of the rotational axis (left–right (LR) axis, up–down (UD) axis, and front–back (FB) axis). Simple effects analysis was conducted when the interaction was significant, and the Bonferroni correction method was used for correction. *p* < 0.05 indicates statistical significance, and the partial Eta square (*η*^2^) represents the effect size of the analysis of variance.

### 3.5 Results

#### 3.5.1 Reaction time

**Figure 5:**
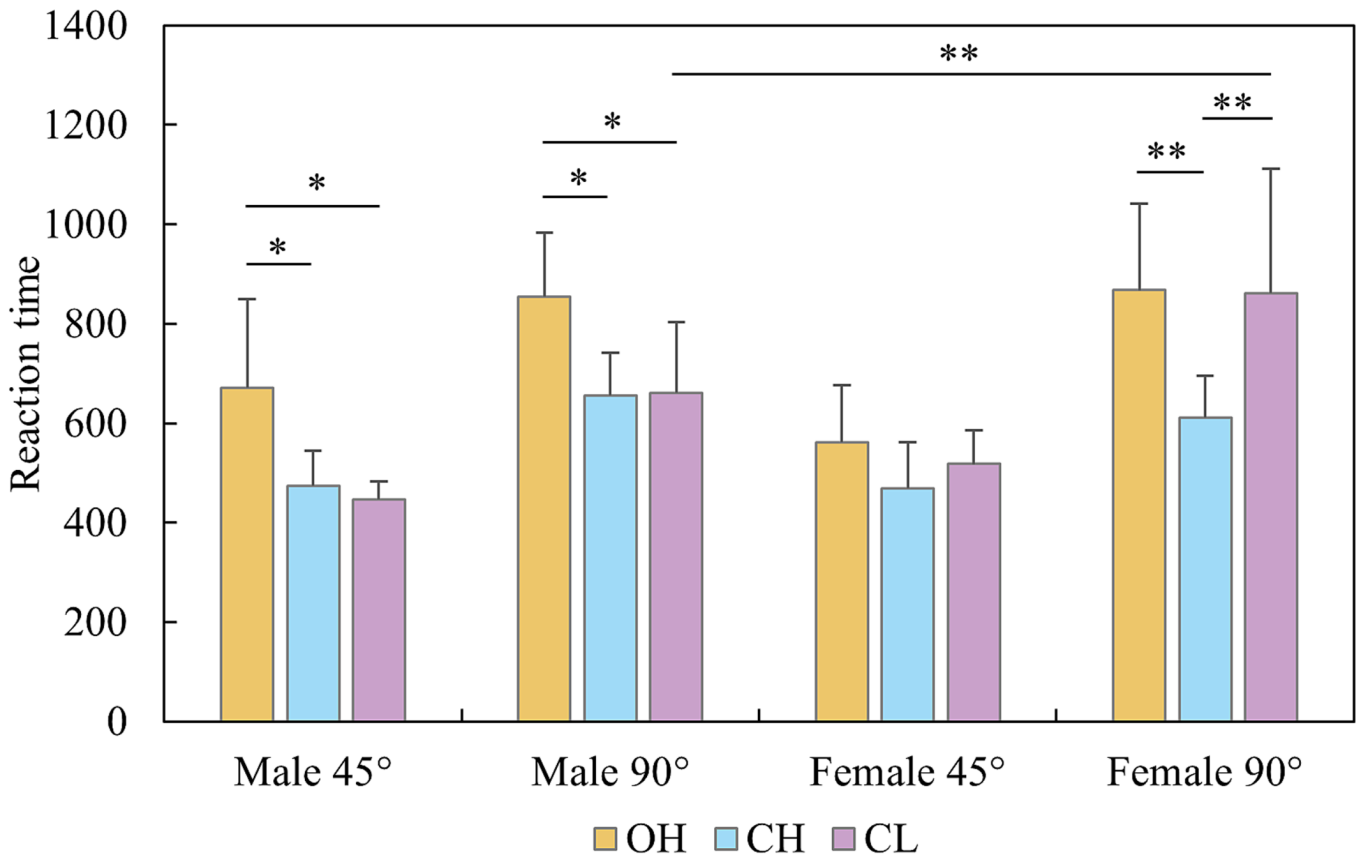
Reaction time of the mental rotation task for group, sex, and angle.

**Figure 6:**
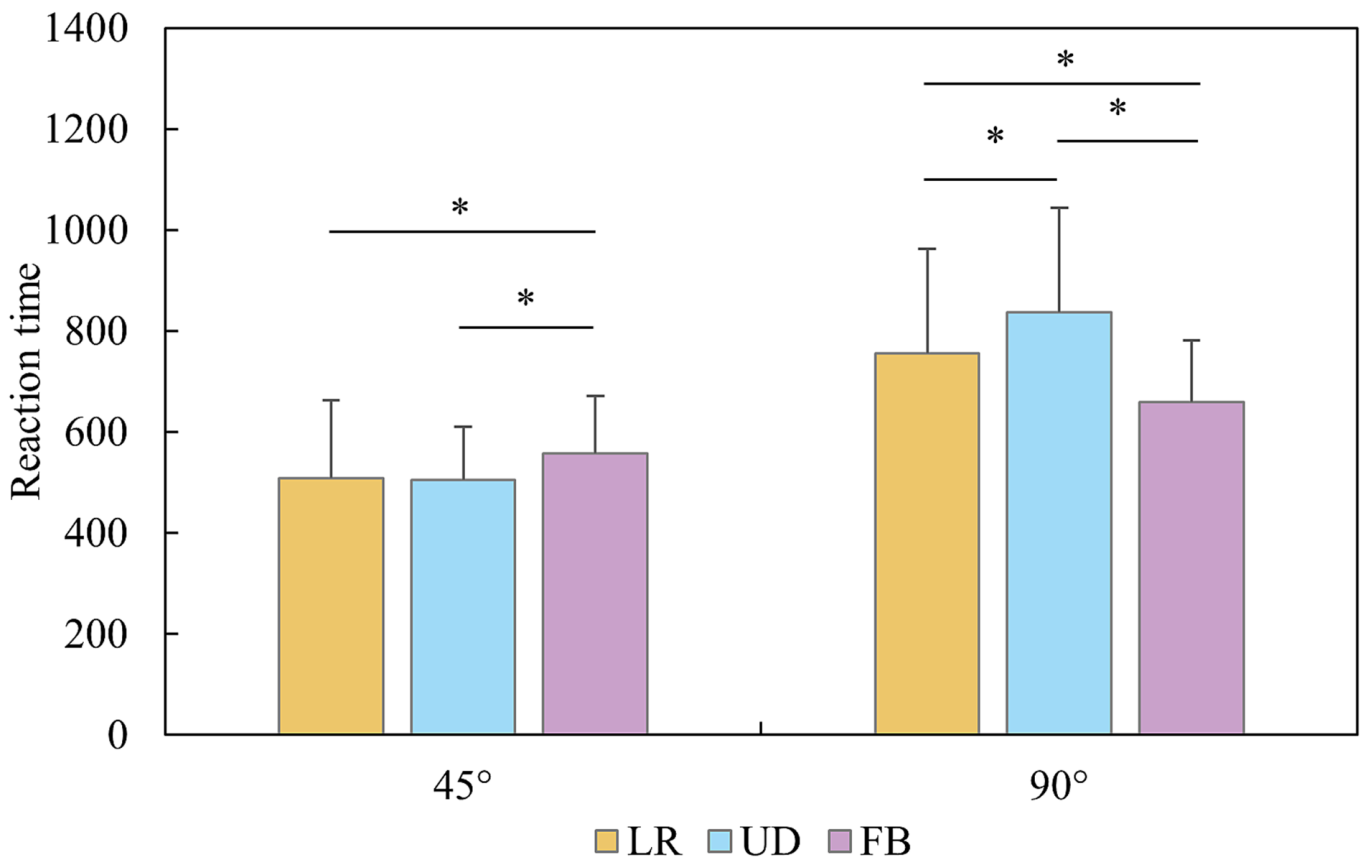
Reaction time of the mental rotation task for axis and angle.

ANOVA revealed significant main effects for group, *F* (2, 40) = 9.382, *p* < 0.001, *η*^2^ = 0.362; rotational axis, *F* (2, 80) = 7.582, *p* < 0.001, *η*^2^ = 0.187; and angle, *F* (1, 40) = 265.224, *p* < 0.001, *η*^2^ = 0.889. The interaction effects for group and angle, *F* (1, 40) = 7.225, *p* = 0.002, *η*^2^ = 0.305; sex and angle, *F* (1, 40) = 6.449, *p* = 0.016, *η*^2^ = 0.163; and rotational axis and angle, *F* (2, 80) = 40.151, *p* < 0.001, *η*^2^ = 0.549, were significant. Additionally, the three-way interaction of group, sex and angle was significant, *F* (2, 40) = 4.385, *p* = 0.020, *η*^2^ = 0.210. Simple effects analysis revealed that male players in the OH group (45° *CI*: 671.76 ± 178.21, 95% *CI* [571.65, 771.88], 90° *CI*: 855.09 ± 129.81, 95% *CI* [728.36, 981.82]) had longer RTs than did those in the CH (45° *CI*: 474.80 ± 69.53, 95% *CI* [404.1, 545.59], 90° *CI*: 655.52 ± 85.73, 95% *CI* [565.91, 745.13]) and CL groups (45° *CI*: 446.97 ± 35.95, 95% *CI* [376.18, 517.76], 90° *CI*: 660.53 ± 143.60, 95% *CI* [570.92, 750.14]) at both angles (*p* < 0.047, for all instances). For female players, the CH group presented a shorter RT than the OH and CL groups did at 90° (*p* < 0.006, for all instances). The OH (867.45 ± 174.42, 95% *CI* [763.97, 970.92]) and CL (861.46 ± 249.52, 95% *CI* [757.99, 964.93]) groups presented longer RTs than did the CH (610.77 ± 84.10, 95% *CI* [514.97, 706.57]) group at 90° (*p* < 0.002, for all instances). Moreover, males (660.53 ± 143.60, 95% *CI* [570.92, 750.14]) demonstrated faster RT than females did (861.46 ± 249.52, 95% *CI* [757.99, 964.93]) at 90° in the CL group (*p* = 0.005). The difference in RT for two angles was significant for every group, axis and sex (*p* < 0.001, for all instances). Interestingly, under 45° conditions, the RT for the LR (508.07 ± 155.28, 95% *CI* [463.10, 553.13]) and UD axes (505.80 ± 106.43, 95% *CI* [474.63, 536.97]) was shorter than that for the FB axis (557.22 ± 114.42, 95% *CI* [521.32, 593.11], *p* < 0.026, for all instances), but under 90° conditions, the RT for the FB axis (660.85 ± 122.48, 95% *CI* [625.82, 695.87]) < LR (756.64 ± 207.49, 95% *CI* [695.33, 817.96]) < UD axis (837.92 ± 207.63, 95% *CI* [785.09, 890.75], *p* < 0.034, for all instances; Figures 5, 6).

#### 3.5.2 Accuracy

**Figure 7:**
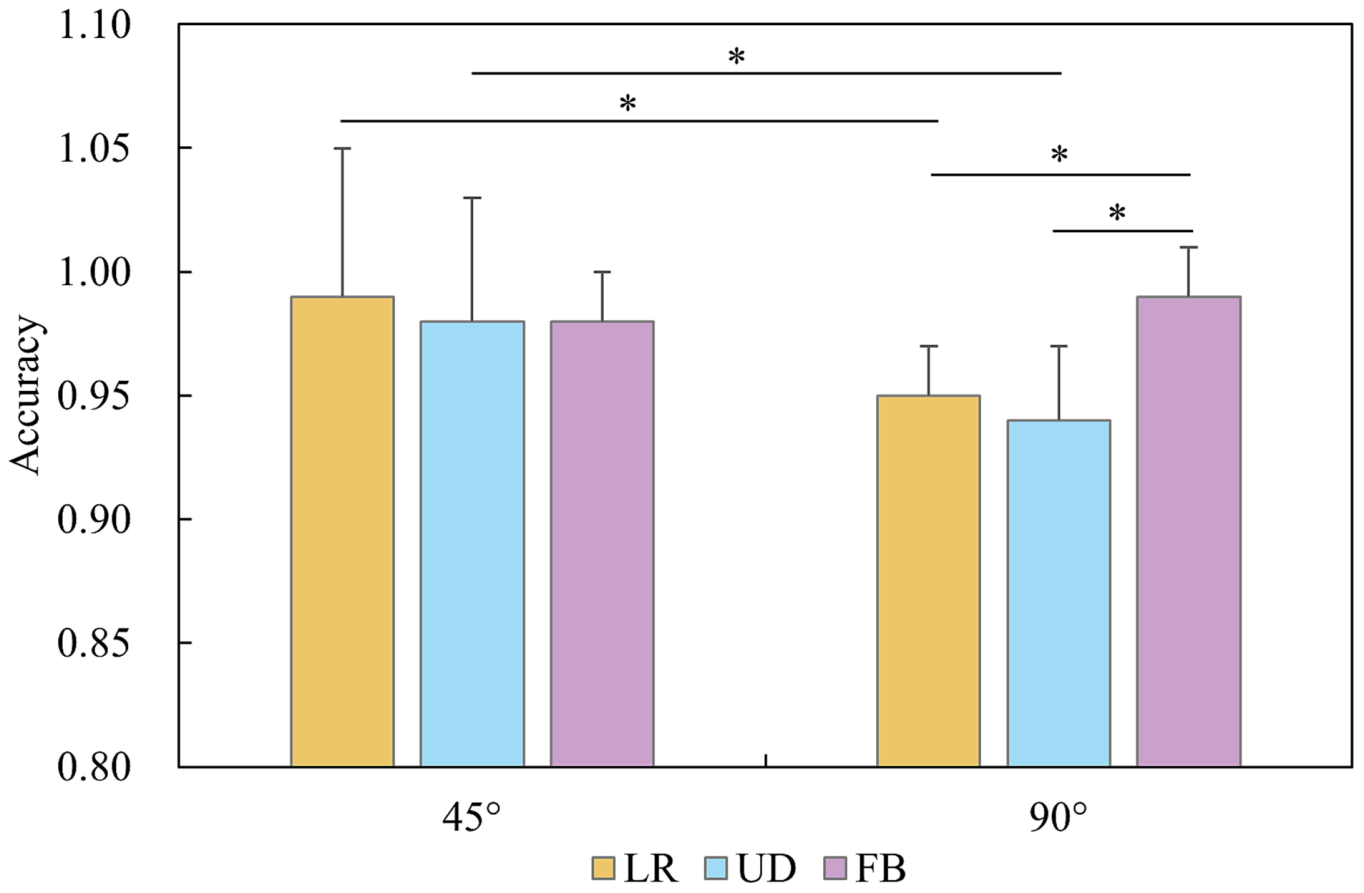
Accuracy of the mental rotation task for group and angle.

ANOVA revealed significant main effects of the rotational axis, *F* (2, 80) = 6.891, *p* = 0.002, *η*^2^ = 0.187, and angle, *F* (1, 40) = 41.577, *p* < 0.001, *η*^2^ = 0.581. The interaction between the rotational axis and angle was significant, *F* (2, 80) = 9.914, *p* < 0.001, *η*^2^ = 0.248. The simple effects analysis revealed that the accuracy at 45° was better than that at 90° for the LR (0.993 ± 0.016, 95% *CI* [0.986, 0.998]) and UD axes (0.980 ± 0.025, 95% *CI* [0.966, 0.994]) but not for the FB axis (0.983 ± 0.018, 95% *CI* [0.976, 0.990], *p* < 0.022, for all instances). Additionally, all the players’ accuracy for the FB axis (0.986 ± 0.015, 95% *CI* [0.980, 0.991]) were superior to those for the LR (0.954 ± 0.059, 95% *CI* [0.927, 0.968]) and UD (0.943 ± 0.049, 95% *CI* [0.925, 0.961]) axes at the 90° (*p* < 0.032, for all instances; Figure 7).

#### 3.5.3 Stage performance

For rotational speed, ANOVA revealed a significant main effect of the rotational axis, *F* (2, 80) = 7.209, *p* = 0.002, *η*^2^ = 0.199, indicating that participants mentally rotated faster for the FB axis (696.77 ± 466.47, 95% *CI* [404.33, 885.27]) than for the UD axis (169.28 ± 86.30, 95% *CI* [124.66, 193.43], *p* = 0.018). The results for the perceptual time revealed a significant main effect of the rotational axis, *F* (2, 80) = 23.809, *p* < 0.001, *η*^2^ = 0.488, indicating that participants took less time to recognize the stimuli for the LR (301.25 ± 216.54, 95% *CI* [223.87, 378.63]) and UD (217.20 ± 131.23, 95% *CI* [165.73, 268.66]) axes than for the FB axis (456.32 ± 155.81, 95% *CI* [397.84, 514.80], *p* < 0.001).

### 3.6 Discussion

In experiment 2, a three-dimensional computerized egocentric MRT with different rotation angles (45°, 90°) was used to test the spatial ability of adolescent athletes in different sport types (open high-spatial sport, closed high-spatial sport and closed low-spatial sport). In general, the results found that under 45° rotational conditions, the reaction time (RT) for the left–right (LR) and up–down (UD) axes were shorter than that for the front-back (FB) axis. Nevertheless, under 90° conditions, the RT for FB < LR < UD, with superior accuracy and rotational speed for the FB axis than for the LR and UD axes. Concerning the effect of gender, male players from the CH and CL groups had shorter RTs than did those from the OH group at both angles. For female players, the CH group presented a shorter RT than the OH and CL groups did at 90°.

The angle effect was confirmed by the result showing that the RT of the large-angle (90°) mental rotation test was significantly longer than that of the small-angle (45°) mental rotation test, and the accuracy of the small-angle test was significantly shorter than that of the large-angle test for the LR and UD axes. This finding is consistent with the study by Bethell-Fox et al., who reported that as the difficulty of the stimulating material increased, the subjects’ responses to the mental rotation test increased (Bethell-Fox and Shepard, 1988). An increase in the rotation angle is clearly associated with an increase in difficulty, suggesting that better mental rotation ability is required for athletes to perform more difficult mental rotation tests and consume more cognitive resources (Zhao et al., 2022).

In terms of the rotation axis, most previous studies carried out paper-plane rotation (i.e., the FB axis in the present study). To scrutinize the MR differences among the three rotational axes in athletes, a study compared the performance of futsal players, rhythmic gymnasts, and sedentary individuals on mental rotation tasks. The research revealed that futsal players had quicker reaction times across all tested planes—frontal, horizontal, and sagittal—than did sedentary players. Notably, their reaction times in the horizontal plane were also faster than those of the rhythmic gymnasts (Fargier et al., 2022). Habacha et al. (2014a) reported that gymnasts were faster than nonathletes in their dominant rotation direction. However, the present study did not find an interaction between sport group and axis. The results demonstrated different mechanisms at different angles. In particular, under 45° rotational conditions, the RT for the LR and UD axes was shorter than that for the FB axis. However, under 90° conditions, the RT for FB < LR < UD, with superior accuracy and rotational speed for the FB axis than for the LR and UD axes. Therefore, when the body rotated a small angle around the UD and LR axes, the postures looked very similar to the standing posture, thus yielding faster RT and perceptual time. However, after large-angle rotation of the body around the UD and LR axes, some body parts were sheltered (i.e., we cannot see the right arm in the 90° UD condition, as well as the feet in the 90° LR condition, see Figure 3), thus increasing the difficulty of the task. In contrast, regardless of the angle at which the body rotates around the FB axis, the body will not be sheltered due to the rotation action, so the results revealed a faster RT and perception speed of the FB axis at a large angle.

After dividing the players into three groups with various spatial characteristics, we investigated the effects of spatial motor experience on mental rotation at small and large angles. Male players from the CH and CL groups had shorter RTs than did those from the OH group at both angles. For female players, the CH group presented a shorter RT than the OH and CL groups did at 90°. The interactions among sport type, sex and angle are very meaningful. According to earlier studies, the CH group should show a significant advantage for MR over the other groups (Jansen and Lehmann, 2013; Schmidt et al., 2015; Feng et al., 2017), which is congruent with our results in females. Nevertheless, how do male players from the CL group, such as runners, obtain better MR ability than those from the OH group, such as basketball and soccer players, and similar MR ability to that of the CH group, such as freestyle skiing players? There are two explanations below. First, the MR for male individuals may benefit much more from sport training, even for closed low-spatial-level sports such as running. Influencing factors may be associated with individual physiological factors such as genetics, lateralization of brain function, sex hormones, and brain size (Siegel-Hinson and McKeever, 2002; Peters et al., 2007). Second, attending sports may lead to better mental rotation ability for boys. A study reported a positive relationship between motor ability and accuracy on mental rotation tasks among primary school-aged and young children (Jansen and Kellner, 2015; Jansen and Heil, 2010).

## 4 General discussion

This study explored the effects of axial rotation on the spatial ability of athletes through two experiments. In Experiment 1, a paper and pencil mental rotation test was conducted, and it was found that the CH group performed better than the other groups did in both embodied and non-embodied tasks, which supported our hypothesis, indicating that skilled sports (i.e., Tai Chi, wrestling, gymnastics, aerobics) have the most significant effects on mental rotation. Moreover, this advantage for the adolescent CH group was also revealed by the computerized MRT in experiment 2, supporting the view of embodiment cognition that all types of cognitive activities are closely related to one’s ability to move the body (Wilson, 2002; Gallese and Sinigaglia, 2011).

Sex and age can affect MR ability. Some studies have shown that sex differences in mental-rotation performance are significant for children under age 13 and increase during adolescence (Voyer, 1995) and sex differences persist in all expert groups, such as STEM, arts, and sports (Tsigeman et al., 2023). Other studies suggest that female athletes can achieve greater improvements in activities; that is, sex differences can be compensated for by participation in physical activities (Habacha et al., 2014b). Researchers believe that women can more significantly modify their visual search behavior during activity participation and thus perform better in the perceptual process or the encoding process of the mental rotation task (Jansen et al., 2012) to increase their mental rotation score. Can the enhancement of mental rotation ability caused by sports activities compensate for the gender differences in mental rotation ability among adolescents? The present study found no sex difference in adolescents’ mental rotation performance for paper and pencil MRTs (in experiment 1), but confirmed a male advantage only in the CL group for computerized MRTs (in experiment 2).

Sex and age can affect MR ability. Some studies have shown that sex differences in mental-rotation performance are significant for children under age 13 and increase during adolescence (Voyer, 1995), and sex differences persist in all expert groups, such as STEM, arts, and sports (Tsigeman et al., 2023). Other studies suggest that female athletes can achieve greater improvements in activities; that is, sex differences can be compensated for by participation in physical activities (Habacha et al., 2014b). Researchers believe that women can more significantly modify their visual search behavior during activity participation and thus perform better in the perceptual process or the encoding process of the mental rotation task (Jansen et al., 2012) to increase their mental rotation score. Can the enhancement of mental rotation ability caused by sports activities compensate for the gender differences in mental rotation ability among adolescents? The present study revealed no sex difference in adolescents’ mental rotation performance for paper and pencil MRTs (in experiment 1) but confirmed a male advantage only in the CL group for computerized MRTs (in experiment 2).

This result offers at least two insights. First, the testing method of mental rotation indeed influences the appearance of gender differences. Compared with computerized MRT, paper-and-pencil testing tends to have less time pressure (Peters, 2005). Under these circumstances, females who participate in sports may receive similar scores to those of males because females can fully understand the questions and effectively manage their time. Second, in Experiment 2, gender differences were observed only in the CL group but not in the other groups, indicating that the spatial characteristics of motor skills can affect gender differences in mental rotation. Specifically, in sports with high spatial transformation (whether open or closed), there was no difference in mental rotation ability between males and females, suggesting that females indeed gain more enhancement during the learning of spatial sports (such as basketball, soccer, gymnastics, etc.). This finding is important for explaining how motor skills influence gender differences in mental rotation and it has certain practical significance. For adolescents, to enhance spatial ability and thereby improve STEM performance, participating in sports with rich spatial transformation in daily physical exercise, school physical education courses, and specialized sports training is a better choice.

The study also has certain limitations. First, concerning the classification of sports, the present study made an initial attempt to categorize sports on the basis of their spatial characteristics. However, the number of sports included in each type of sports project is relatively small. Future research should include more sports of the same type for comparison. Second, with the advancement of cognitive neuroscience, the relationship between motor skills and mental rotation ability can now be explored within the field of brain plasticity. For example, if spatial sports training indeed leads to greater improvements in mental rotation ability for females, it is necessary to determine whether this process is due to differences in brain processing (Feng and Li, 2021; Yin, 2024). To address this question, more studies utilizing electroencephalography (EEG) or functional near-infrared spectroscopy (fNIRS) technology are needed.

## 5 Conclusion

The present study utilized spatial factors and sex to assess the mental rotation ability of adult and adolescent athletes and, for the first time, confirmed the role of the rotational axis in the relationship between sport expertise and mental rotation in light of embodied cognition. The results showed that the CH group and OH group outperformed the CL group in the non-embodied task and the CH group was better than the other groups in the embodied and tasks. Under 45° rotational conditions, the RT for the LR and UD axes was shorter than that for the FB axis. However, under 90° conditions, the RT for FB < LR < UD, with superior accuracy and rotational speed for the FB axis than for the LR and UD axes. Male players from the CH and CL groups had shorter RTs than did those from the OH group at both angles. For female players, the CH group presented a shorter RT than the OH and CL groups did at 90°. No sex difference was found for paper and pencil MRTs, and a male advantage existed only in the CL group for computerized MRTs. These results suggest that the motor skills associated with axial rotation could promote MR performance and compensate for sex differences in MR ability.

## Data Availability

The original contributions presented in the study are included in the article/Supplementary material, further inquiries can be directed to the corresponding author.
